# Dr. W. O. Rodgers

**Published:** 1898-04

**Authors:** 


Dr. W. O. Rodgers, of Omaha, Nebraska, (Medical Department, University of Buffalo, '83,) died at his home March 10, 1898. Dr. Rodgers was examiner-in-chief of the Woodmen of the World and had been identified with fraternal insurance orders during the past ten years. He had secured an extensive professional practice and died lamented by a large acquaintance.

